# Comparative study between ventriculosubgaleal shunt and external ventricular drain for management of post infective hydrocephalus among pediatrics

**DOI:** 10.1007/s00381-024-06344-5

**Published:** 2024-03-05

**Authors:** Abdelaziz Abdelhamid Ismail, Ahmed Nageeb Taha, Hatem Ibraheem Badr, Ahmed Zaher, Samy Abbas Elbaz, Amr Farid Khalil

**Affiliations:** 1 Faculty of Medicine, Neurosurgery Department, Mansoura University, Mansoura, Egypt; 2 Faculty of Medicine, General Surgery Department, Mansoura University, Mansoura, Egypt

**Keywords:** Post infective hydrocephalus, Meningitis, Intracranial pressure, Ventriculosubgaleal shunt, External ventricular drain

## Abstract

**Purpose:**

Post infective hydrocephalus (PIH) is a type of hydrocephalus which occurs after an infection of the brain or cerebrospinal fluid (CSF). Treatment of PIH requires temporary measures such as external ventricular drain (EVD) and ventriculosubgaleal shunt (VSGS) until CSF becomes clear and ready to implement VP shunt.

Limited research has been done to explore the tradeoff between these approaches particularly in pediatric PIH patients.

Our study compares the complications, mortality rates, and the cost of used resources of both procedures.

**Methods:**

A prospective study was conducted for 18 months in which we compared between VSGS and EVD for management of PIH involving 42 randomized cases with 21 patients in group A operated by VSGS and 21 patients in group B operated by EVD.

**Results:**

Our results show a statistically significant difference between both groups in the duration of implementation of VSGS/EVD until resolution of infection occurs. Additionally, a higher rate of pediatric intensive care unit (PICU) admission and a longer length of hospital stay (LOS) were recorded among the EVD group. No statistically significant difference between the number of complications that happened in both despite variations in their forms. Moreover, both groups showed nearly similar mortality rates.

**Conclusion:**

There is no significant difference in the rate of complications between VSGS and EVD for PIH. Based on that, VSGS emerges as a favorable and cost-effective option for the management of PIH which leads to less economic burden on patients and the country’s health resources, especially in developing countries.

## Introduction

Hydrocephalus (HCP) is a critical issue particularly in pediatric neurosurgery. HCP is an active distension of the ventricular system of the brain resulting from inadequate passage of CSF from its point of production within the cerebral ventricles to its point of absorption into the systemic circulation. When hydrocephalus occurs following cerebral pyogenic or chronic infections, it is referred to as “post-infective” hydrocephalus (PIH). In rare condition, HCP might happen following fungal or viral infections of the brain [1, 2].

In PIH cases, highly proteinaceous CSF leads to impaired CSF flow making ventriculoperitoneal shunt more prone to blockage and infection and also causes irritation of the peritoneum leading to ileus. The cost of shunt procedures and revisions is high compared to temporizing procedures. Delaying the shunt procedure may lead to better survival and subsequent reduced number of repeat surgeries and costs [3–5].

Transient interventions for PIH encompass both intermittent and continuous cerebrospinal fluid drainage approaches. Intermittent CSF drainage techniques are utilized for both analysis and treatment of hydrocephalus and may consist of serial lumbar punctures, serial trans fontanelle aspiration, or placement of a transcutaneous tappable reservoir. Conversely, steady CSF drainage could be accomplished by using an external ventricular drain (EVD) or the formation of a ventriculosubgaleal shunt (VSGS) [6, 7].

VSGS consists of a shunt tube with one end in the lateral ventricles while the other end is inserted into the subgaleal space of the scalp which is a fibroareolar layer found between the galea aponeurotica and the periosteum of the scalp. Subgaleal space can be used as a shunt to drain excess CSF from the ventricles due to its elastic and absorptive capabilities this avoids iatrogenic infection risk associated with EVD or the risk of developing porencephalic cysts associated with repeated anterior fontanelle ventricular taps. Additionally, it is more physiologic as it is not associated with fluid or electrolyte loss [12].

EVD is a catheter inserted percutaneously into the ventricular system and connected to an external strain gauge transducer. EVDs allow for both measurement of ICP and therapeutic drainage of CSF. EVD insertion is considered the most accurate and cost-effective method for ICP monitoring. For EVD, we can easily rule outraised ICP. However, for VSGS, it is more difficult to determine the functionality of the device based on clinical evaluation alone without CT brain as palpation for the consistency of the subgaleal pocket is highly subjective [13].

So, we performed this study to manage PIH via VSGS or EVD to assess the efficacy of both procedures.

## Patients and methods

This prospective study was conducted for 18 months and included 42 pediatric patients with post infective hydrocephalus who were operated on in Mansoura University Hospitals.

We excluded patients with hydrocephalus due to other causes not including infection or patients older than 18 years old. The included patients were divided into two groups according to the surgical approach performed. Group A comprised 21 cases who were operated on by VSGS and group B comprised 21 patients who were operated on by EVD. Simple randomization will be done through computer-generated numbers.

Every patient was subjected to history taking that comprised name, age, gender, residence, consanguinity, and family history of similar conditions. The examinations included vital signs, heart rate, and respiratory rate. The neurological examination included head circumference, anterior fontanelle parameters, cranial nerve examination, and ocular motility examination. The radiological investigations included cranial US, CT, and MRI of the brain, while the the laboratory investigations included CSF analysis weekly.

**Fig. 1:**
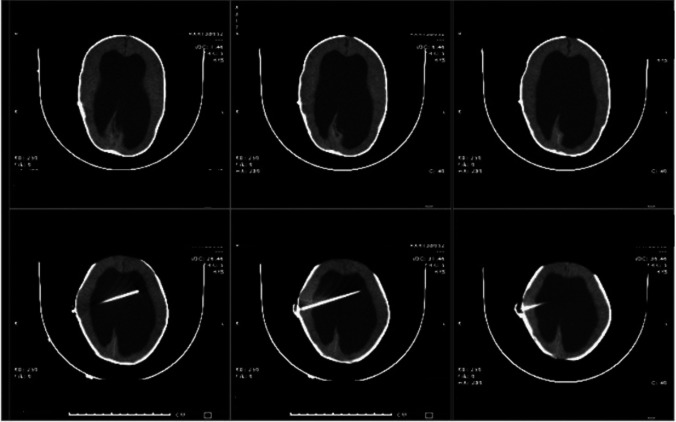
16-month-old infant with VPS infection and hydrocephalus

**Fig. 2:**
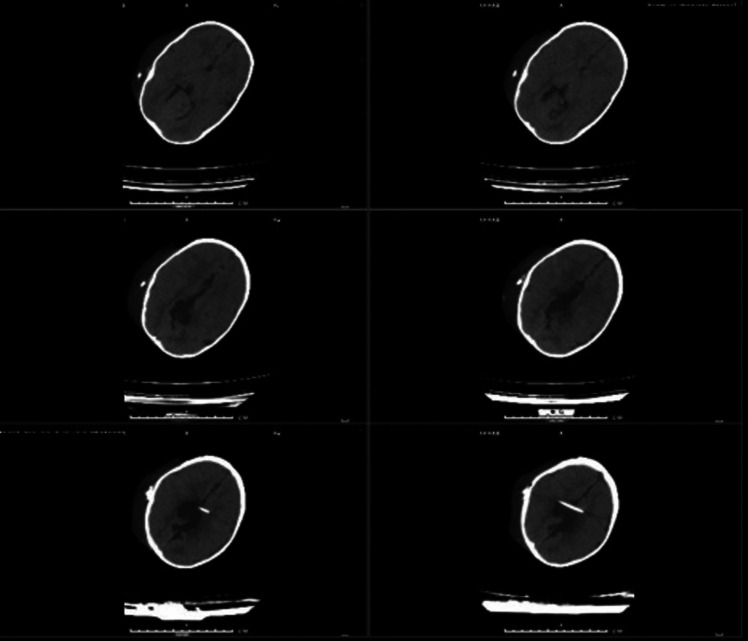
16-month-old infant after removing VPS and insertion of VSGS

All patients were assessed for PIH and were managed by VSGS or EVD and assessment of improvement either clinically or radiologically besides noting the disadvantages and advantages of each procedure (Figs. 1 and 2). Follow-up for patients were done.

## Statistical analysis

Data were analyzed by utilizing SPSS version 28. Qualitative data were presented as number and percent, quantitative data were tested for normal distribution by Kolmogorov–Smirnov test after that described as mean ± SD for normal distribution of data and median and range for non-normal distribution of data. The proper statistical test was done based on data type with the next recommended tests; chi-square, Student *t* test, and Mann–Whitney *U* test.

## Results

Table 1 shows that the median age of group A is 11 months ranging from 3 months to 10 years versus 17 months ranging from 7 months to 14 years for group B without statistically significant difference between them (*P* = 0.127). Males represent 57.1% of group A versus 66.7% of group B without statistically significant difference between them (*P* = 0.525). Regarding causes of procedure, there was no statistically significant difference between studied groups in terms of causes of procedure. For group A; 57.1% post meningitis, 33.4% post VPS infection, and 9.5% post ETV infection, and for group B; 42.9% post meningitis, 42.9% post VPS infection, and 14.2% post ETV infection. Regarding the duration of implementation of VSGS/EVD (days) until resolution of infection occurs, the median duration was 52 days ranging from 3 to 85 days for group A versus 29 ranging from 2 to 48 days for group B with statistically significant difference between groups (*P* = 0.001).

**Table 1:** Comparison of demographic characteristics, causes of procedure, and duration of VSG/EVD (days) of the studied groups

	**Group A** ***N* = 21(%)**	**Group B** ***N* = 21(%)**	**Test of significance**
**Age/years** Median (min–max)	11 months (3 months–10 years)	17 months (7 months–14 years)	*Z* = 1.53 *P* = 0.127
**Sex**			
Male Female	12 (57.1) 9 (42.9)	14 (66.7) 7 (33.3)	${\chi }^{2}$ = 0.404 *P* = 0.525
**Causes of procedure**			
Post VPS infection Post meningitis Post ETV infection	7 (33.4) 12 (57.1) 2 (9.5)	9 (42.9) 9 (42.9) 3 (14.2)	${\chi }^{2}$= 0.879 *P* = 0.644
**Duration of VSG/EVD (days)** Median (min–max)	52(3–85)	29(2–48)	Z = 3.2 *P* = 0.001*

Table 2 shows no statistically significant difference between the studied groups as regard complications incidence. The most frequent complications were Blockage and pouch collapse and devitalized wound and CSF leak.

**Table 2:** Comparison of complications between studied groups

	**Group A** ***n* = 21(%)**	**Group B** ***n* = 21(%)**	**Test of significance**
**Blockage and pouch collapse**	6 (28.6)	3 (14.3)	${\chi }^{2}$= 1.27 *P* = 0.259
**Devitalized wound and CSF leak**	5 (23.8)	2 (9.5)	${\chi }^{2}$= 1.54 *P* = 0.214
**Subdural collection**	3 (14.3)	1 (4.8)	FET = 1.11 *P* = 0.606
**Slipped**	0	2 (9.5)	FET = 2.10 *P* = 0.487
**Bone remodeling**	1 (4.8)	0	FET = 1.02 *P* = 1.0

Table 3 demonstrates a statistically significant higher frequency of PICU admission among group B than group A (80.9% versus 23.8%, respectively). Also, a higher median hospital stay duration is detected among group B than group A (35 days versus 5 days for groups B and A, respectively). As regard, cases need revision; 6 cases (28.6%) of group A versus 10 cases (47.6%) of group B need revision. Also, there was no statistically significant difference between the studied groups as regard mortality rates with 33.3% of group A versus 38.1% of group B died.

**Table 3:** Comparison of management and mortality between studied groups

	**Group A** ***n* = 21(%)**	**Group B** ***n* = 21(%)**	**Test of significance**
**PICU admission**	5 (23.8)	17 (80.9)	${\chi }^{2}$= 13.75 *P* = 0.002*
**Hospital stay/days** **Median (min–max)**	5 (3–21)	35 (2–52)	*Z* = 5.42 *P* < 0.001*
**Cases need revision**	6 (28.6)	10 (47.6)	${\chi }^{2}$= 1.62 *P* = 0.203
**Mortality**			
Dead	7 (33.3)	8 (38.1)	${\chi }^{2}$= 0.104 *P* = 0.747

## Discussion

HCP is an abnormal intraventricular collection of CSF in the brain caused by an increase in the rate of CSF formation or a reduced rate of CSF absorption. The management might be challenging [8, 9].

In the context of PIH cases, proteinaceous CSF leads to reduction of CSF flow making (VPS) more prone to blockage [10]. A lot of therapeutic approaches are used which include temporary ways such as EVD, Ommaya reservoir, recurrent lumbar punctures, or VSGS. Every therapeutic method has its benefits and drawbacks; selecting the proper approach depends on various parameters, which include environmental, medical, and patient factors [11].

We compared between VSGS and EVD for management of PIH in a study involving 42 randomized cases (*n* = 21 per group), group A, 21 patients operated by VSGS and group B, 21 patients operated by EVD. No significant differences were observed in age, gender distribution, or procedural causes between the two groups. Complications incidence did not significantly differ.

In harmony with our findings, Kitchen and his colleagues found that infection has been considered the main complication of EVD up to 45% and this was accompanied by significant morbimortality, prolonged hospital stay, and increased financial burden [14]. Also, Amen et al. demonstrated the rate of infections and exposure in 20% of VSGS cases. VSGS obstructions and migrations were recorded in about 6% of their patients [12]**.**

Sil and his colleagues recorded that blockage and infection were observed in 15% and 2%, respectively, of their 215 participants who had hydrocephalus of various causes, such as the PIH who underwent VSGS [10]. Even though certain researches recorded mild infection frequencies following VSGS (about 5%) [15], different researches recorded a higher incidence frequency (47.6%) [16]. It is important to consider that most of these researches were performed in the post hemorrhagic hydrocephalus patients instead of the PIH ones, aside from one study that included PIH cases and recorded a higher frequency in comparison with our study. In addition, it is of great importance to consider that the operation was conducted in an already contaminated field [16].

Our study showed that the median duration was 65 days for group A versus 33 for group B with significant difference between the 2 groups. Amen et al. showed that the average duration of VSGS was 35 days [12]. Such duration was in accordance with the one recorded by Tubbs et al. in 2003 which had an average value of 37 days [21, 17]. Different researches recorded more prolonged durations of 56 days, as recorded by Kariyattil and his colleagues applying VSGS in PIH cases [16]. Additionally, Kutty et al. study revealed an average duration of 40 days (range 20–60 days) until resolution of infection occurs [20]. An essential factor which enhances durability is the absorptive capacity of the subgaleal space and the formation of generous spaces during the dissection process [18].

The current study demonstrated a statistically significant higher frequency of PICU admission among group B than group A (80.9% versus 23.8%, respectively). Also, a higher median hospital stay duration was detected among group B than group A (35 days versus 5 days for groups B and A, respectively). Hypothetically, EVD has been demonstrated to be accompanied by a higher possibility of infection in comparison with the VSGS, as EVD makes the CSF subjected to the external environment, and that was formerly verified by a lot of researches. Therefore, it leads to a consequent increase in the LOS due to the need for drain repositioning along with the increase in morbidities among ill neurosurgical patients [17, 19].

On the other hand, Elzain et al. in 2022 evaluated 35 children (in Sudan) who were managed by EVD due to different cranial conditions. They have revealed that one-third were admitted to the PICU. The longest LOS was 61 days with the mean duration of 3 weeks. Therefore, cases with EVD are favorably nursed in the ICU to avoid the device-associated complications, which include slippage, over-drainage, and different medical non-neurosurgical side effects [13].

No significant difference was detected between the current studied groups in terms of revision frequency: 6 cases (28.6%) of group A versus 10 cases (47.6%) of group B need revision. Amen et al. found that 16 cases with VSGS (32%) needed shunt revision [12]**.**

The current study illustrated no statistically significant difference between studied groups in terms of mortality rates with 33.3% of cases died in group A versus 38.1% in group B. The primary cause of mortality was disease progression leading to uncontrolled infection and septicemia. Kutty et al. study revealed that mortality was observed in 44.4% of patients with PIH [20]. Conversely, Elzain et al. in 2022, ten patients who underwent EVD (*n* = 10/35, 28.6%) died; five secondary to cardiac arrest; four secondary to sepsis; and one secondary to respiratory arrest [13].

Amen et al. showed that mortality was encountered in 4 cases (8%) owing to sepsis [12]. Death rates after VSGS range from 9 to 20%, and the majority of such cases died due to different complications instead of shunt-related ones [20].

Amen et al. reported that VSGS provides a lot of benefits compared to other transient approaches in PIH cases. It is not accompanied by fluid or electrolyte loss. Additionally, the closed drainage system does not expose the CSF to the external environment which reduces the risk of infection. In addition, the time of the surgical approach was short, and most of the patients were followed at the outpatient clinic. This was accompanied by a reduction in the LOS and a decrease in socio-economic burden [12].

Additional data from different institutions are now required to elaborate on our results in such conditions. Data on the rates of infections associated with VSGS and EVD placement in developing nations are scant.

## Limitations of the study

The small sample size has been considered the main limitation. Also, all cases were from a single-based center that cannot be generalized to the overall populations. In addition, the absence of long-term follow-up has been considered another limitation. Such limitations must be well handled in the future researches. Further large-scale investigations and studies on a large number of individuals are essential.

## Conclusion

Our findings showed no significant difference in the rate of complications between VSGS and EVD for PIH. However, the use of VSGS had a lower frequency of intensive care unit (ICU) admissions and a reduced length of hospital stay (LOS) compared to EVD. VSGS emerges as a favorable and cost-effective option for the management of PIH which leads to less economic burden on patients and the country’s health resources, especially in developing countries.

## Data availability

No datasets were generated or analysed during the current study.

## Declarations

Ethical approval and consent to participate.

Approval from IRB, Faculty of Medicine, Mansoura University was obtained before the study and written informed consent was taken from all subjects before their participation in the study. Confidentiality and privacy were respected. The right to leave the study at any time was guaranteed for all operated subjects.

## References

[1] Afifi AM, Abdullah JM, Siregar JA, Idris Z (2019). A retrospective study on the first cerebrospinal fluid taken from external ventricular drainage insertion in meningitis patients with hydrocephalus. Malays J Med Sci.

[2] Amen MM, Zaher A, Badr HI, Elshirbiny MF, Elnaggar AM, Khalil AF (2022). Ventriculosubgaleal shunt as a proposed technique for post-infectious hydrocephalus. Childs Nerv Syst.

[3] Chatterjee S (2018) Post-infective hydrocephalus. pediatric hydrocephalus 1–30

[4] Chatterjee S, Chatterjee U (2011). Overview of post-infective hydrocephalus. Childs Nerv Syst.

[5] Di Rocco C (2006) Cinalli G, Maixner WJ, Sainte-Rose C (eds): Pediatric hydrocephalus. Child’s Nerv Syst 22(2):204–205

[6] Eid S, Iwanaga J, Oskouian RJ, Loukas M, Jerry Oakes W, Tubbs RS (2018). Ventriculosubgaleal shunting-a comprehensive review and over two-decade surgical experience. Childs Nerv Syst.

[7] Elzain MA, Ahmed MM, Salim AD (2022). External ventricular drainage: indications and outcome among Sudanese Children. Sudan J Paediatr.

[8] Garg S, Kulkarni S, Deopujari CE, Biyani N (2021). Study of neurodevelopmental outcome in patients with non-tumoral hydrocephalus with shunt surgery done in infancy. J Pediatr Neurosci.

[9] Hochstetler A, Raskin J, Blazer-Yost BL (2022). Hydrocephalus: historical analysis and considerations for treatment. Eur J Med Res.

[10] Ihara S (2022). Ommaya reservoir and the external ventricular drainage. No Shinkei Geka.

[11] Karimy JK, Reeves BC, Damisah E, Duy PQ, Antwi P, David W, Wang K, Schiff SJ, Limbrick DD, Alper SL, Warf BC, Nedergaard M, Simard JM, Kahle KT (2020). Inflammation in acquired hydrocephalus: pathogenic mechanisms and therapeutic targets. Nat Rev Neurol.

[12] Kariyattil R, Mariswamappa K, Panikar D (2008). Ventriculosubgaleal shunts in the management of infective hydrocephalus. Childs Nerv Syst.

[13] Kitchen WJ, Singh N, Hulme S, Galea J, Patel HC, King AT (2011). External ventricular drain infection: improved technique can reduce infection rates. Br J Neurosurg.

[14] Kutty RK, Sreemathyamma SB, Korde P, Prabhakar RB, Peethambaran A, Libu GK (2018). Outcome of ventriculosubgaleal shunt in the management of infectious and non-infectious hydrocephalus in pre-term infants. J Pediatr Neurosci.

[15] Nagy A, Bognar L, Pataki I, Barta Z, Novak L (2013). Ventriculosubgaleal shunt in the treatment of posthemorrhagic and postinfectious hydrocephalus of premature infants. Childs Nerv Syst.

[16] Nee LS, Harun R, Sellamuthu P, Idris Z (2017). Comparison between ventriculosubgaleal shunt and extraventricular drainage to treat acute hydrocephalus in adults. Asian journal of neurosurgery.

[17] Omar MA, Mohd Haspani MS (2010). The risk factors of external ventricular drainage-related infection at hospital kuala lumpur: an observational study. Malays J Med Sci.

[18] Padayachy L, Ford L, Dlamini N, Mazwi A (2021). Surgical treatment of post-infectious hydrocephalus in infants. Childs Nerv Syst.

[19] Sil K, Ghosh SK, Chatterjee S (2021). Ventriculo-subgaleal shunts-broadening the horizons: an institutional experience. Childs Nerv Syst.

[20] Thomale UW, Cinalli G, Kulkarni AV, Al-Hakim S, Roth J, Schaumann A, Bührer C, Cavalheiro S, Sgouros S, Constantini S, Bock HC (2019). TROPHY registry study design: a prospective, international multicenter study for the surgical treatment of posthemorrhagic hydrocephalus in neonates. Childs Nerv Syst.

[21] Tubbs RS, Smyth MD, Wellons JC, Blount J, Grabb PA, Oakes WJ (2003). Life expectancy of ventriculosubgaleal shunt revisions. Pediatr Neurosurg.

